# Deep Learning Approach for Assessing Air Quality During COVID-19 Lockdown in Quito

**DOI:** 10.3389/fdata.2022.842455

**Published:** 2022-04-04

**Authors:** Phuong N. Chau, Rasa Zalakeviciute, Ilias Thomas, Yves Rybarczyk

**Affiliations:** ^1^School of Information and Engineering, Dalarna University, Falun, Sweden; ^2^Grupo de Biodiversidad Medio Ambiente y Salud, Universidad de Las Américas, Quito, Ecuador

**Keywords:** air pollution, machine learning, deep learning - artificial neural network (DL-ANN), data-driven modeling and optimization, COVID-19

## Abstract

Weather Normalized Models (WNMs) are modeling methods used for assessing air contaminants under a business-as-usual (BAU) assumption. Therefore, WNMs are used to assess the impact of many events on urban pollution. Recently, different approaches have been implemented to develop WNMs and quantify the lockdown effects of COVID-19 on air quality, including Machine Learning (ML). However, more advanced methods, such as Deep Learning (DL), have never been applied for developing WNMs. In this study, we proposed WNMs based on DL algorithms, aiming to test five DL architectures and compare their performances to a recent ML approach, namely Gradient Boosting Machine (GBM). The concentrations of five air pollutants (CO, NO_2_, PM_2.5_, SO_2_, and O_3_) are studied in the city of Quito, Ecuador. The results show that Long-Short Term Memory (LSTM) and Bidirectional Recurrent Neural Network (BiRNN) outperform the other algorithms and, consequently, are recommended as appropriate WNMs to quantify the effects of the lockdowns on air pollution. Furthermore, examining the variable importance in the LSTM and BiRNN models, we identify that the most relevant temporal and meteorological features for predicting air quality are Hours (time of day), Index (1 is the first collected data and increases by one after each instance), Julian Day (day of the year), Relative Humidity, Wind Speed, and Solar Radiation. During the full lockdown, the concentration of most pollutants has decreased drastically: −48.75%, for CO, −45.76%, for SO_2_, −42.17%, for PM_2.5_, and −63.98%, for NO_2_. The reduction of this latter gas has induced an increase of O_3_ by +26.54%.

## Introduction

In recent years, millions of deaths around the world have been caused by the polluted environment due to toxic emissions from industries, traffic, and the growing human population (Piqueras and Vizenor, 2016; WHO, 2021a,b). Among the most common atmospheric pollutants are carbon monoxide (CO), nitrogen oxides (NO and NO_2_), sulfur dioxide (SO_2_), ozone (O_3_) and particulate matter (PM), predominantly fine particulate matter (with aerodynamic diameter ≤ 2.5 μm, PM_2.5_). These pollutants, at certain established concentration levels, can damage respiratory and cardiovascular systems (Pope et al., 2012; Lelieveld et al., 2015). Furthermore, recent studies have shown that bad air quality could aggravate the symptoms of the coronavirus disease 2019 (COVID-19) (Gardiner et al., 2020; Wu et al., 2020).

In 2020, while the COVID-19 spread throughout the world, Ecuador was one of the most affected countries, with 50,183 confirmed cases and 4,199 deaths, reported on June 21st (WHO, 2020). To reduce the disease spread, the country implemented the first exceptionally strict lockdown between March 15 and June 2, 2020, and from thereon, has been progressively relaxing the security measures (until September 2020)^1^. This situation makes Quito, the capital city of Ecuador, an excellent case study to assess the effects of different levels of lockdown.

The simplest approach to assessing the impact of the COVID-19 lockdowns on air quality is to compare the average concentration of a pollutant before and during the lockdown (Gkatzelis et al., 2019; Zalakeviciute et al., 2020). More advanced methods consist of developing a model to predict the pollution level assuming a business as usual (BAU) scenario and quantifying the lockdown effects. Air quality prediction is traditionally based on the application of atmospheric chemical transport models (CTMs), which provide a mathematical framework for the description of emission patterns, meteorology, chemical transformations, and removal processes (Seinfeld and Pandis, 2016). Such CTMs can be combined with higher resolution dispersion models to provide local air quality levels in street canyons (Gidhagen et al., 2021). More recently, statistical methods, such as Machine Learning (ML) (Barré et al., 2021; Betancourt et al., 2021; Lovrić et al., 2021; Nitheesh et al., 2021; Rybarczyk and Zalakeviciute, 2021), have proven their efficiency and reliability in predicting the concentrations of pollutants in the atmosphere.

Meteorological normalization is a method that uses meteorological features to predict the concentrations of air contaminants under BAU conditions (Grange et al., 2018). Since we take into account the actual meteorological condition during the lockdown, it allows us to obtain Weather Normalized Models (WNMs). So far, the WNMs have been built from ML algorithms, such as Random Forest and Gradient Boosting Machine (GBM). Grange et al. (2018) proposed WNMs based on Random Forest algorithm for PM_10_ analysis. Afterwards, Rybarczyk and Zalakeviciute (2021) and Barré et al. (2021) used GBM for developing the WNMs to quantify the effects of COVID-19 lockdowns on air quality.

More recently, DL algorithms have shown a better performance than ML on several predicting problems. For example, Convolution Neural Network (CNN) fits well for image and signal processing (LeCun et al., 1998; Qin et al., 2019). Long-Short Term Memory (LSTM) is more adapted for time series prediction (Kuremoto et al., 2014; Ong et al., 2016) and natural language processing (NLP) (Hochreiter and Schmidhuber, 1997). Bidirectional Recurrent Neural Network (BiRNN) is mainly applied for timeseries data (Li et al., 2019), signal processing (Schuster and Paliwal, 1997), automated translation (Sundermeyer et al., 2014), NLP (Liwicki et al., 2007), and bioinformatics (Pollastri and McLysaght, 2005). In addition to that, the architecture of each DL method can be customized to improve model accuracy. In recent years, DL has received much attention for developing air quality prediction models. Recurrent Neural Network (RNN) has been applied for air quality monitoring (Kristiani et al., 2020) and air quality classification (Fan et al., 2017; Zhao et al., 2018). CNN has been used for air pollution index prediction and NO_2_ estimation (Ragab et al., 2020). Meanwhile, LSTM was applied for predicting CO, NO_2_, O_3_, PM_10_, SO_2_ and pollen concentrations in Madrid (Navares and Aznarte, 2020), and for modeling air quality in India (Krishan et al., 2019). Finally, some studies used DL for predicting air quality with BiRNN (Tong et al., 2019), Gated Recurrent Unit (GRU) (Athira et al., 2018) and multi-source data for forecasting PM_2.5_ (Sun et al., 2021). Considering that DL has the potential to provide higher accuracy, we propose DL-based WNMs by testing five DL architectures. Another motivation for using a DL-based approach is its modeling flexible, such as new data can be included in the training without having to scan the whole dataset.

This paper aims to develop WNMs based on DL for studying the effect of COVID-19 on air quality. The accuracy of GBM and five DL algorithms is compared to identify the best models for predicting air pollution under BAU conditions. The objective is to obtain a more accurate assessment of the effect of the lockdowns (strict and relaxing) on air quality, using the capital city of Ecuador as a case study. Five representative pollutants of the urban air quality are studied: NO_2_, SO_2_, CO, O_3_, and PM_2.5_. Afterwards, we use SHapley Additive exPlanations (SHAP)^2^ to discover the feature importance of the inputs for the best model, which can reveal the interrelations between predictors and pollutant concentrations. Finally, the impacts of the COVID-19 lockdown are quantified by calculating the difference between the real and predicted values of the pollutant concentrations under the BAU assumption.

In summary, the main goals of this study are outlined as follows:

- Developing WNMs based on DL to estimate air pollution for the five most representative urban pollutants.
- Assessing the impacts of the COVID-19 lockdowns on air quality in Quito by selecting the best WNMs.
- Identifying the feature importance for the best WNMs and analyzing the relationship between temporal, meteorological features, and air pollutants.

The remainder of this paper is organized into three sections. Section Materials and Methods includes a description of the site and instrumentation, data collection, data processing, and implemented methods. Section Results and Discussions presents the results and discussion. Our conclusions and future work are presented in the final section.

## Materials and Methods

### Site Description and Instrumentation

The Ecuadorian capital, Quito, is located in South America right on the equator. The climate is mild and stable in terms of daily temperature variations, with wet (September–May) and dry (June–August) seasons (Zalakeviciute et al., 2018a). It is a high elevation city in the Andes mountains at 2,850 meters above mean sea level (m.a.s.l.), housing a population of 2.2 million people (EMASEO, 2011; INEC, 2011). Due to the reduced availability of oxygen (70%) at this altitude (Andes mountains), and poor-quality diesel and gasoline, the city is known for its long-term air pollution problems (Zalakeviciute et al., 2018a,b).

The city successfully manages a long-term air quality monitoring network, functioning in accordance with the requirements of the Environmental Protection Agency of the United States (U.S. EPA) (Secretaria de Ambiente, dd). The study site - Belisario (m.a.s.l 2,835 m, coord. 78°29'24” W, 0°10'48” S) is in a central traffic-busy district and is the best representative of the capital city of Ecuador.

Air quality monitoring instruments were set on the patio of a local school. A ThermoFisher Scientific 48i instrument was used to acquire the concentrations of CO (EPA method No. RFCA-0981-054). A ThermoFisher Scientific 43i was used for SO_2_ (EPA method No. EQSA-0486-060). ThermoFisher Scientific 49i was used for O_3_ (EPA method No. EQOA-0880-047). ThermoFisher Scientific 42i was used for NO_2_ (EPA method No. RFNA-1289-074). Finally, Thermo Scientific FH62C14-DHS was used to obtain PM_2.5_ concentrations (EPA No. EQPM-0609-183). Apart from the air quality data, meteorological parameters were also measured in the same monitoring station. For that, a complete automatic weather station was used. Wind speed and direction were measured using a MetOne instrument. Relative humidity, temperature and precipitation were measured by Thies Clima equipment. Finally, a Kipp Zonen radiometer measured solar radiation, and Vaisala equipment measured atmospheric pressure.

### Data

The data include meteorological, temporal variables, and five air pollutant concentrations. The seven meteorological features are: Solar Radiation (SR), Wind Direction (WD), Wind Speed (WS), Atmospheric Pressure (p), Precipitation (Prec), Temperature (T) and Relative Humidity (RH). The four temporal variables are Julian Day (or Day of the Year), Weekday, Hours (or Time of Day), and Index (the index is started from 1 January 2016 and incremented by one at each instance). These temporal variables are additional variables in WNMs, not directly affecting the atmospheric concentration, but reflecting cyclic emission patterns. Hours account for emissions at rush hours. The Julian Day is a periodic term that represents seasonal emissions. The Weekday reflects the difference in human mobility between weekends and weekdays. The Index variable is denoted as a trending feature. The predicted features are the concentrations of NO_2_, SO_2_, CO, O_3_, and PM_2.5_.

The dataset includes 4 years and 9 months of hourly data between 2016 and 2020. Instances with empty values for certain features are eliminated from the cleaned dataset. Afterwards, we divided the data into three parts. The first part is the training set, from 1 January 2016 to 15 January 2020 (2 months before the COVID-19 lockdown). The second part, which is the testing set, is from 16 January 2020 to 15 March 2020 (the day of the national lockdown). In WNMs, the months before the application of interventions are commonly used for the testing set (Petetin et al., 2020; Barré et al., 2021; Rybarczyk and Zalakeviciute, 2021). The third part is the full lockdown (from 16 March to 1 June 2020) and partial lockdown (from 2 June to 30 September 2020) periods, which is used to quantify the change of pollutant concentration, through the best WNM models.

**Figure 1:**
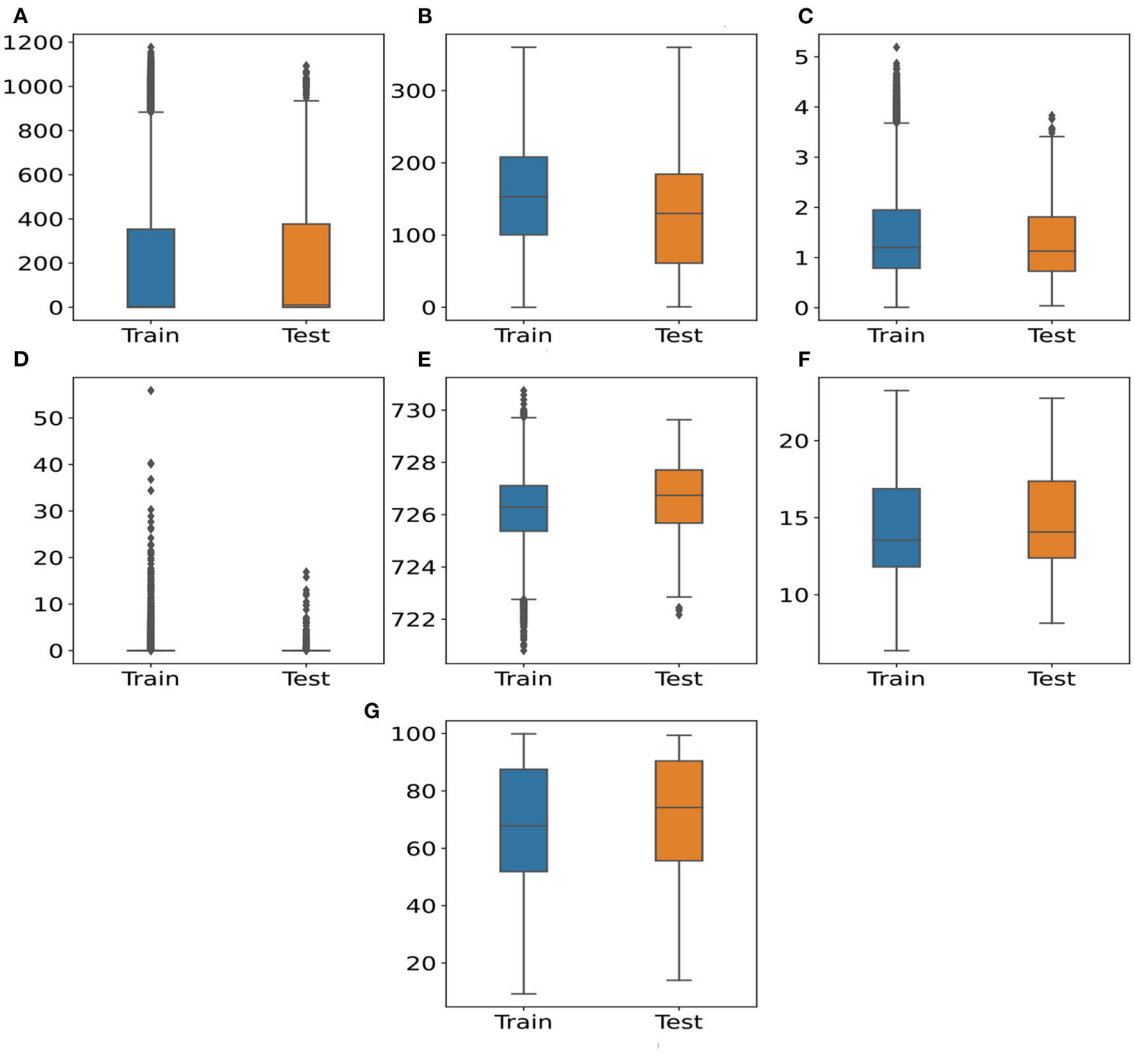
Distributions of meteorological features between training and testing set. **(A)** SR (W/m^2^) **(B)** WD (°) **(C)** WS (m/s) **(D)** Prec (mm) **(E)** p (mb) **(F)** temperature (°C) **(G)** RH (%).

Figure 1 illustrates the distribution of the seven meteorological features for the training and testing sets. From this figure, we can identify three groups. The first group of variables indicates that the median of testing data is higher than that on training data (Figures 1A,E–G). On the contrary, the second group includes WS and WD (Figures 1B,C). The median of these features in testing sets is lower than the median in the training data. The last group consists of Prec features, where the boxes are small in both training and testing sets (Figure 1D). This feature is skewed, which can be caused by the weather characteristics in Quito with wet and dry seasons. Overall, most distributions of the meteorological features on the training sets are able to cover the distributions of the meteorological features on the testing set.

**Figure 2:**
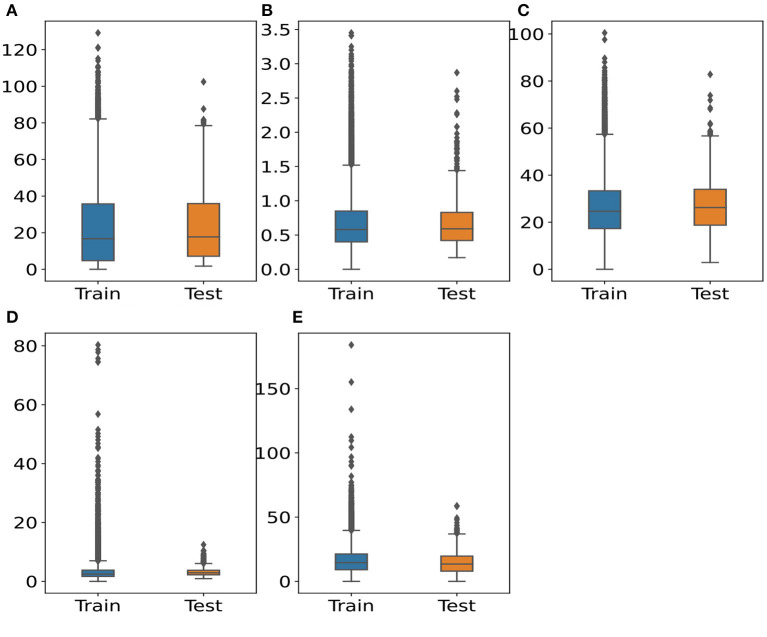
Distributions of pollutant concentrations between training and testing set. **(A)** O_3_ (μg/m^3^) **(B)** CO (mg/m^3^) **(C)** NO_2_ (μg/m^3^) **(D)** SO_2_ (μg/m^3^) **(E)** PM_2.5_ (μg/m^3^).

Figure 2 depicts the distributions of five pollutant concentrations. There are several outliers in the data. The outliers consist of data points that are higher than 1.5 times the interquartile range (IQR). Meanwhile, outlier levels in air pollutants should be different from 1.5^*^IQR (Schmid et al., 2000). Additionally, it can be seen that the median and height of the boxes in training and testing data are sharply similar. This observation allows us to retain all original pollutant data to develop WNMs and assess the effects of COVID lockdowns.

### Method

**Figure 3:**
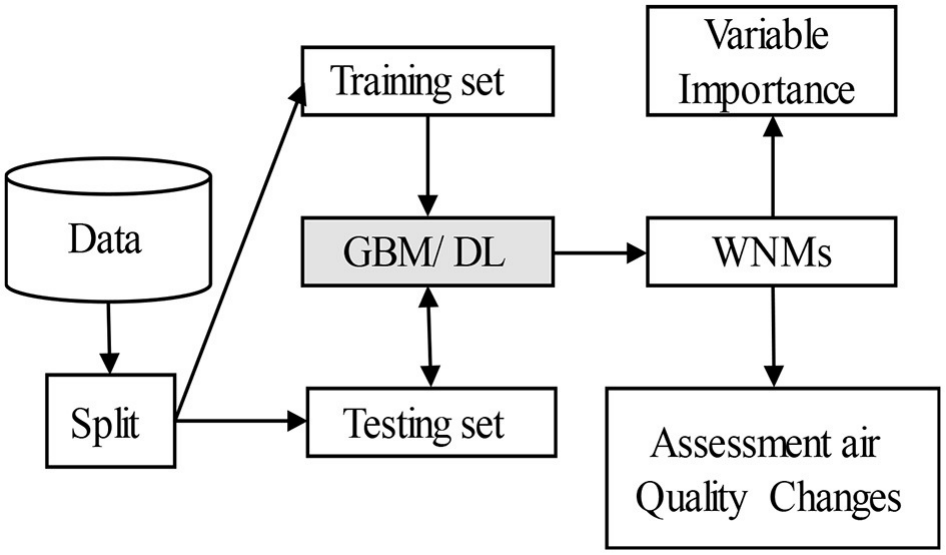
Workflow of the data analysis.

Figure 3 represents an overview of our research method. First, we eliminated empty values and split the data into training and testing sets. Second, we used the training and testing data to develop and evaluate the performance of GBM and DL models. The architecture and implementation of the GBM and DL models are described in Sections Gradient Boosting Machine and DL Models. The experimental setups describe how to tune the parameters of GBM and DL algorithms (Section Experimental Setups). Third, all models are evaluated to select the best WNMs, based on the two metrics explained in Section Evaluation Metrics. Afterwards, the best WNMs are used for “Variable Importance” and “Assessment of Air Quality Changes”. The respective importance of each feature for the best WNMs is obtained by using the SHAP values method (Section SHapley Additive exPlanations for Model Explanation). Finally, the best WNMs is used to quantify the pollution change in Quito during the COVID-19 lockdown. The predicted values from the best WNMs are considered as pollution levels of contaminants under BAU conditions. The air quality changes are the differences between the predicted and the actual values during the lockdown periods.

#### Gradient Boosting Machine

GBM is a powerful method of decision tree-based ensemble learning (Friedman, 2001). It was used in the previous study for assessing the effect of the COVID-19 lockdown on air quality (Barré et al., 2021; Rybarczyk and Zalakeviciute, 2021). The generalization of the algorithm is a stage-wise additive model of *n* individual regression trees following the algorithm presented in Table 1. GBM sequentially builds regression trees for all the data set features in a fully distributed way, which means that the trees are built in parallel. At each iteration from 1 to M, the instructions defined at line 2(a–d) are repeated *K* times. Equation (1) is used to obtain the outputs.


(1) $$\begin{array}{r} \hat{f}\left(x\right)=\;\overset{N}{\underset{n=1}{\sum }}{\;\hat{f}}^{n}\left(x\right) \end{array}$$

**Table 1:** GBM procedure in H_2_O library.

Initialize *f*_*k*0_ = 0, *k* = 0, 1, …, *K*
For m = 1 to M: 1. Set $p_{k}\left(x\right)=\frac{e^{f_{k}\left(x\right)}}{\overset{K}{\underset{l=1}{\sum }}\left(e^{f_{l}\left(x\right)}\right)},k=1,2,\ldots ,K$ 2. For k=1 to K: a. Compute *r*_*ikm*_ = *y*_*ik*_−*p*_*k*_(*x*_*i*_), *i* = 1, 2, …, *N* b. Fit a regression tree to the targets: *r*_*ikm*_, *i* = 1, 2, …, *N*, giving terminal regions *R*_*jim*_, *j* = 1, 2, …, *J*_*m*_ c. Compute $\gamma_{ikm}=\frac{K-1}{K}\frac{\underset{x_{i}\in R_{jkm}}{\sum }\left(r_{ijk}\right)}{\underset{x_{i}\in R_{jkm}}{\sum }|\left(r_{ikm}\right)|\left(1-|\left(r_{ikm}\right)|\right)},j=1,2,\ldots ,J_{m}$ d. Update $f_{km}\left(x\right)=f_{k,m-1}\left(x\right)+\overset{J_{m}}{\underset{j=1}{\sum }}\left(\gamma_{jkm}I\right)\left(x\in R_{jkm}\right)$
Output: $\hat{f}\left(x\right)=f_{kM}\left(x\right),k=1,2,\ldots ,K$

#### DL Models

In recent years, five kinds of DL methods (CNN, LSTM, RNN, BiRNN, and GRU) have been widely used in the literature (LeCun et al., 1998; Ong et al., 2016; Athira et al., 2018; Jogin et al., 2018; Qin et al., 2019; Tong et al., 2019; Kristiani et al., 2020; Navares and Aznarte, 2020). Each of these algorithms has its advantages and drawbacks. For this reason, the proposed approach aims to compare their performance for air quality prediction. Once the best model is identified, it is used to assess pollution changes caused by the different levels of lockdown.

**Figure 4:**
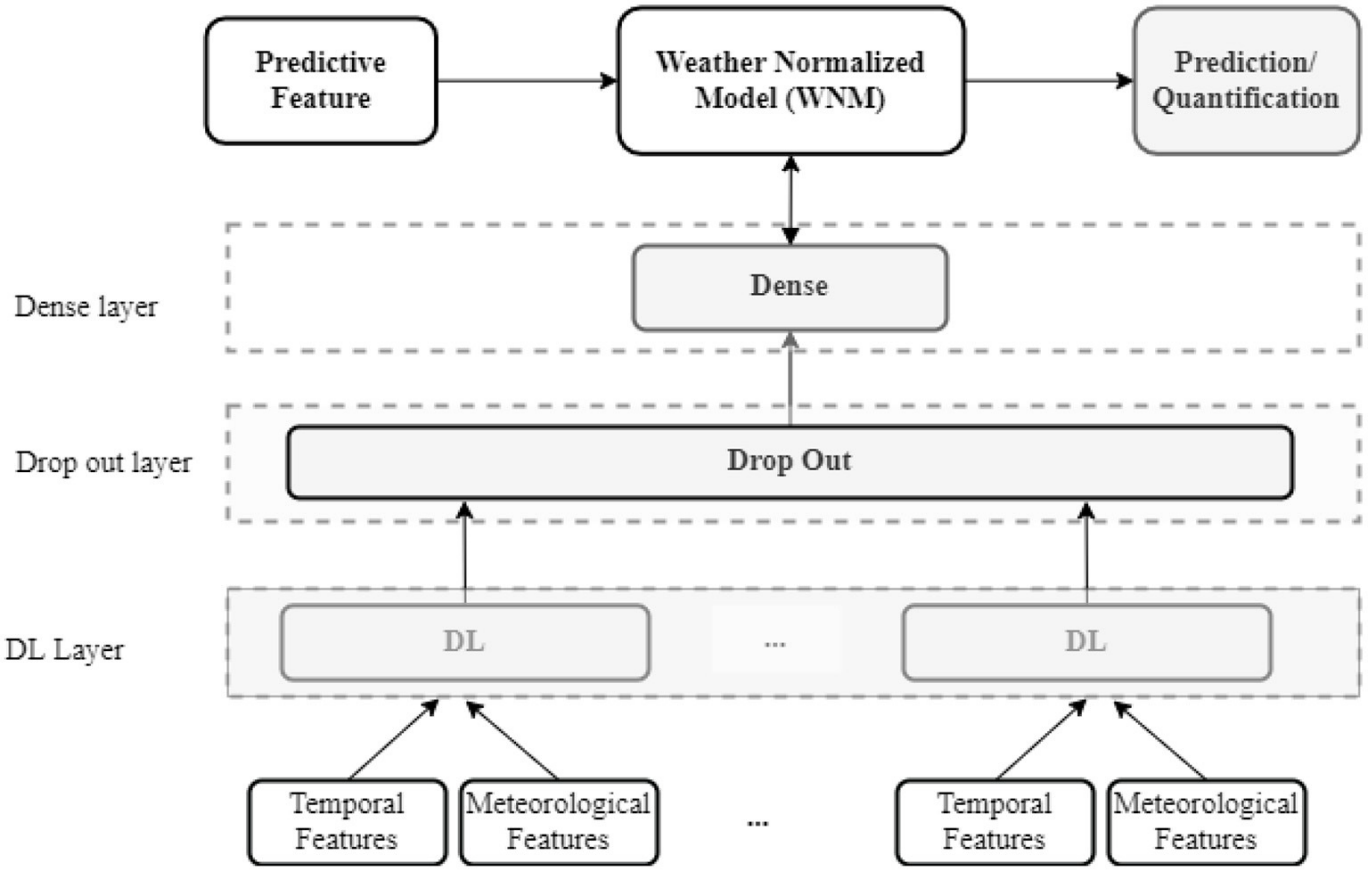
The model with general DL layer.

The proposed DL models are based on the five layers presented in Figure 4. First, an input layer adapts the temporal and meteorological features to the DL layer. This layer transforms the original data into three-dimensional data with Min-Max Scaler based on the number of features and the number of “Timesteps.” Second, the DL layer is set up for capturing the characteristics of the data. CNN, LSTM, RNN, BiRNN and GRU are used to compare the performance of each of the DL architectures. Third, a “Drop out” layer is introduced after the DL layer to reduce the risk of over-fitting. Since the outputs are real numbers, a Dense layer is added afterwards to adapt the output of the “Drop out” layer to the predicted targets. Then, the weights of the WNM are adjusted from the predictive feature to obtain the best model. Although more complex DL models could be implemented (e.g., CNN-LSTM or LSTM-LSTM), the intended scope of this study is to focus on a DL layer for a fair comparison between the DL architectures.

The architectures, advantages, and disadvantages of five DL methods (CNN, LSTM, RNN, BiRNN, and GRU) are described in detail in the rest of this section.

##### Convolution Neural Network

**Figure 5:**
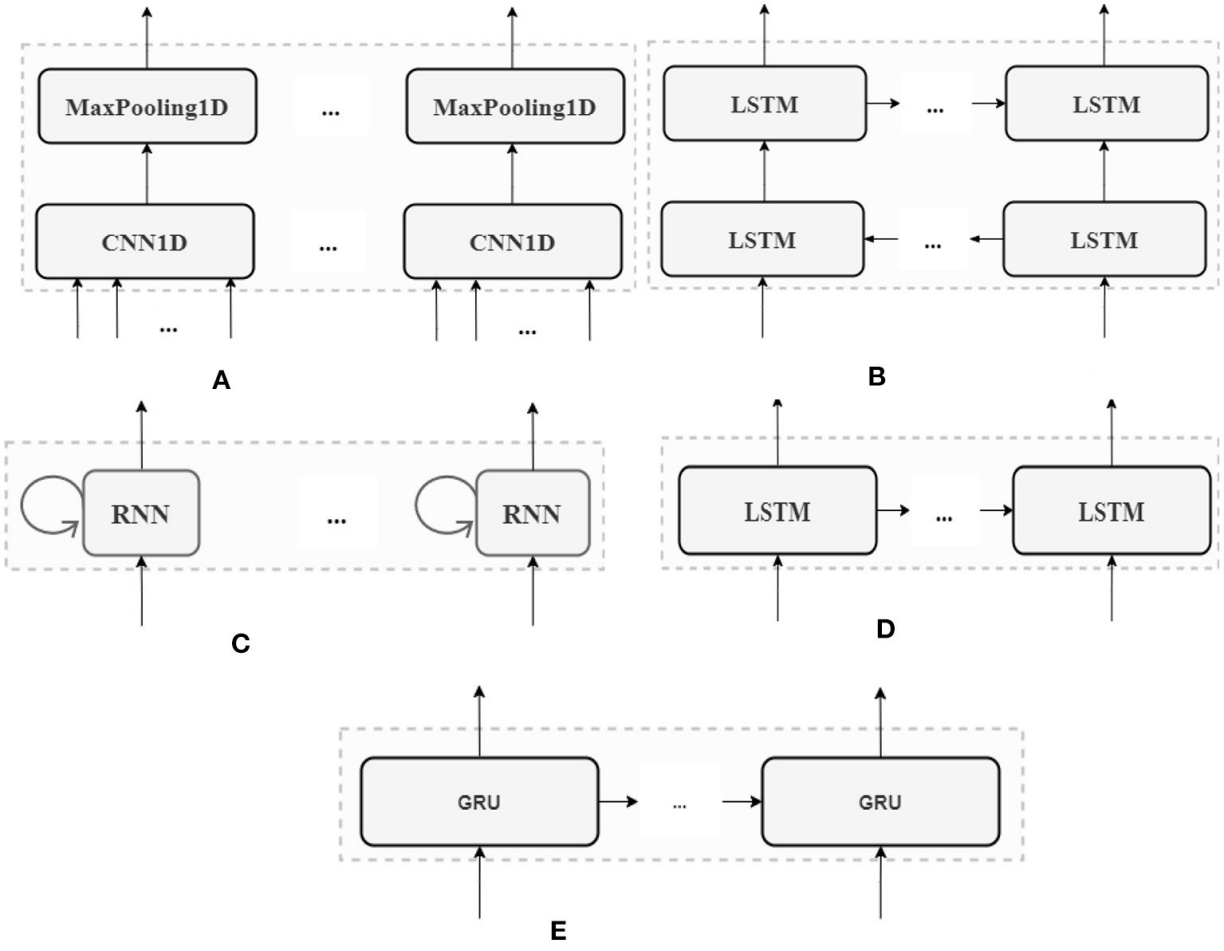
Five DL architectures. **(A)** CNN1D layer **(B)** BiRNN layer **(C)** RNN layer **(D)** LSTM layer **(E)** GRU layer.

There are two basic types of CNN architecture: CNN1D and CNN2D. While the former can be used for sequence data, the latter is applied to image or high dimensional data. Figure 5A shows the connections inside the CNN1D layer. The CNN1D cells receive and learn from the inputs. Afterwards, the outputs of CNN1D are sent to the MaxPooling1D cells. The MaxPooling1D cells decrease the number of parameters to learn and support the internal presentations in the CNN1D layer. The outputs from the MaxPooling1D cells are sent to the “Drop Out” layer. In the CNN1D layer, there is no connection between the CNN cells.

##### Recurrent Neural Network Based Architectures

In the RNN architecture, instead of being connected to each other, the cells are connected to the former sequence of the input itself (Figure 5C). For example, at time t, the RNN cells link to the previous state of themselves at the time (*t*−1). There are many ways of using RNN efficiently. For the sake of a fair comparison between the different DL architectures tested here, only one-layer RNN is used as in Figure 5C.

The LSTM layer is used in the same layer as CNN or RNN. However, the LSTM cells work differently. The LSTM has two outputs at time t. The first output is connected to the “Drop Out” layer in the DL model. The second output forwards information to the next LSTM cell, and the cell also receives the input from the previous LSTM cell (Figure 5D).

BiRNN combines two hidden LSTM layers, and each LSTM layer has an opposite direction. Hence, the BiRNN can get backward or forward information (Figure 5B). This is a powerful DL architecture for sequence data with bidirectional context. However, BiRNN is slower than LSTM, because it runs in both forward and backward recursion (Li et al., 2019).

GRU is a new version of LSTM with fewer gates inside. The gates can keep or reject the information from the inputs. Additionally, the internal structure of GRU is simpler, and its performance is faster than LSTM because it has fewer computational operations to update the parameters of the hidden layers. In our model, the GRU layer is similar to the LSTM layer, with LSTM cells replaced by GRU cells (Figure 5E).

#### Experimental Setups

We conducted the experiments on Dell Precision 7550. The computer has 16 CPUs with Intel Core I7-10875H@2.3 GHz, with 8 cores and 128 GB memory. Additionally, it includes Nvidia Quadro T2000 with 4 GB memory. Since the DL models require GPU devices, our experiments used the whole memory of the Nvidia card on the computer with the “Timesteps” = 12. The configuration of each method is described in the rest of this section.

##### GBM Tuning

GBM is implemented using the H_2_O library for Python. The parameters of the model are shown in Table 2. We set the tuning of “ntrees” parameters by maximizing 15,000 trees. The learning rate was tuned to 0.05, in order to satisfy the convergence criterion quickly. If the GBM model cannot improve, the model will stop after the “stopping_round” iterations. The other parameters were used as the sample in the H_2_O library. These parameters are similar to a previous study (Rybarczyk and Zalakeviciute, 2021).

**Table 2:** Parameters for GBM in H_2_O library.

**Parameters**	**Values**
Learning rate	0.05
Balance_classes	True
ntrees	15,000
stopping_round	10

##### DL Tuning

All DL models are run with the TensorFlow library (version 2.3.0) on Python programming language. The parameters for all the DL models are listed in Table 3. We tuned “Timesteps” and “The number of nodes” to find the best model for each pollutant. “Epochs” is a parameter used as a condition for stopping the model. Specifically, the DL stops after 300 iterations if no global optimization is reached. Otherwise, the Early Stopping Strategy is applied, based on the “Patience” parameter. This means that the training model finishes after 20 iterations (Patience = 20) if the performance cannot improve. The “Drop out” (0.25) eliminates 25% of connections to reduce the overfitting. The learning rate is 0.05, which increases the speed of convergence in DL models. “Batch size” controls the gradient error in the models. CNN requires two additional parameters, which are the kernel size and polling size. If “Timesteps” is one, these parameters are one, and they should be tuned to three if “Timesteps” is greater or equal to three. A total of 30 models (five “Timesteps” × six “The number of nodes”) for each DL architecture were created.

**Table 3:** Parameters for all DL models.

**Parameters**	**Values**
Timesteps	1, 3, 6, 9, 12
The number of nodes	16, 32, 48, 64, 96, 128
Patience	20
Drop out	0.25
Loss function	mse
Learning_rate	0.05
Batch size	500
Epochs	300
Kernel size (only CNN)	1,3
Polling size (only CNN)	1,3

#### Evaluation Metrics

Two metrics were used to evaluate and compare the performance of the GBM and WNMs (DL-based models): Root Mean Square Error (RMSE) and coefficient of determination (*R*^2^). RMSE and *R*^2^ were computed according to Equations (2) and (3), respectively. RMSE ranges from zero to plus infinity, and *R*^2^ ranges from 0 to 1. In both equations, y_i_ are predicted values and y_i_ are actual values of sample *i*, and *p* is the size of the testing set; $\overset{\bar{\;}}{y}$ are mean of actual values. To obtain the best evaluation, the RMSE must be as close as possible to zero and the *R*^2^ as close as possible to one.


(2) $$\begin{array}{r} \text{RMSE }=\sqrt{\frac{\overset{p}{\underset{i\;=\;1}{\sum }}{\left(\;{\hat{y}}_{i}\;-\;y_{i}\right)}^{2}}{p}} \end{array}$$

(3) $$\begin{array}{r} {\text{R}}^{2}\;=\;1-\frac{\overset{p}{\underset{i\;=\;1}{\sum }}{\left({\hat{y}}_{i}\;-\;y_{i}\right)}^{2}}{\overset{p}{\underset{i\;=\;1}{\sum }}{\left(\overset{\bar{\;}}{y}\;-\;y_{i}\right)}^{2}} \end{array}$$

### SHapley Additive exPlanations for Model Explanation

DL has been applied in many research areas, because of its high performance. On the other hand, DL is considered as a black box, which makes it difficult to explain why a model has a good prediction. Nevertheless, the important values for input features can be disclosed by using SHAP (Lundberg and Lee, 2017). This method is based on Equation (4). In this equation, φ_*j*_*(val)* is the SHAP value for feature *j*; when a feature has higher φ_*j*_*(val)*, it is assessed as a stronger contributor for the model; *S* is the set of features in the model or predictors; *p* is the number of input variables; *x*_*j*_ is the vector values of feature *j*; and *val(S)* is the output variable with the set *S* or pollutant concentrations.

SHAP values are used for interpreting the correlations between the input and output features. The higher the value is, the higher the importance is. SHAP values can provide a deeper understanding of the contribution of meteorological and temporal variables to pollutant concentrations.


(4) $$\begin{array}{l} \phi_{\text{j}}\left(\text{val}\right)\text{=​​​​​​}\sum_{\text{S}\subseteq \{{\text{x}}_{\text{1}},\ldots ,{\text{x}}_{\text{p}}\}\backslash \{{\text{x}}_{\text{j}}\}}^{\;}\text{​}\text{​​​​​​​​}\frac{\left|\text{S}\right|!\left(\text{p-}\left|\text{S}\right|\text{-1}\right)!}{\text{p}!}\left(\text{val}\left(\text{S}\cup \{{\text{x}}_{\text{j}}\}\right)\text{-val}\left(\text{S}\right)\right) \end{array}$$

## Results and Discussions

In Section Performance, the performance of five DL architectures and GBM is compared, based on the lowest RMSE and highest *R*^2^. In order to look into the black boxes, the variable importance for the best WNMs is carried out in Section Variable Importance. Finally, these latter models are used for assessing the effects of COVID-19 lockdowns on air quality in Quito (Section Quantifying Air Quality Changes).

### Performance

The performance of WNMs before the lockdown is presented in Table 4. Overall, the accuracy of the proposed DL models is better than that of the GBM. Particularly, BiRNN and LSTM outperform the GBM algorithm for predicting pollution concentrations. While BiRNN yields the best results with respect to O_3_ concentrations (RMSE = 7.1854; *R*^2^ = 0.8628), NO_2_ concentrations (RMSE = 7.3644; *R*^2^ = 0.5772) and CO concentrations (RMSE = 0.1830; *R*^2^ = 0.7148), the LSTM gives the best results with respect to SO_2_ (RMSE = 1.0506; *R*^2^ = 0.4702) and PM_2.5_ concentrations (RMSE = 6.7677; *R*^2^ = 0.4310). Consequently, three BiRNNs and two LSTMs models are used for the second part of the study, which consists of identifying variable importance and assessing the impacts of COVID-19 lockdown on air quality.

**Table 4:** Performance of GBM and five DL models for evaluation period.

			**Methods**					
**Pollutant**	**Data**	**Metric**	**CNN**	**LSTM**	**RNN**	**BiRNN**	**GRU**	**GBM**
O_3_	Train	RMSE	11.3565	8.1623	10.2825	**7.8713**	7.8576	7.4504
		*R* ^2^	0.6817	0.8356	0.7391	**0.8471**	0.8476	0.8630
	Test	RMSE	10.2842	7.2843	8.9215	**7.1854**	7.5269	7.9034
		*R* ^2^	0.7189	0.8590	0.7885	**0.8628**	0.8494	0.8340
NO_2_	Train	RMSE	10.0527	8.2206	9.0477	**8.4251**	7.7267	7.2283
		*R* ^2^	0.3276	0.5504	0.4553	**0.5277**	0.6028	0.6524
	Test	RMSE	8.8637	7.3883	7.9328	**7.3644**	7.5262	8.2571
		*R* ^2^	0.3875	0.5744	0.5094	**0.5772**	0.5584	0.4685
CO	Train	RMSE	0.2942	0.2288	0.2663	**0.2344**	0.2456	0.2149
		*R* ^2^	0.4274	0.6539	0.5308	**0.6366**	0.6009	0.6944
	Test	RMSE	0.2436	0.1893	0.2263	**0.1830**	0.1983	0.2242
		*R* ^2^	0.4945	0.6949	0.5638	**0.7148**	0.6652	0.5720
PM_2.5_	Train	RMSE	8.7046	**7.7477**	8.3617	7.9143	8.1093	8.2775
		*R* ^2^	0.2587	**0.4127**	0.3159	0.3872	0.3566	0.3297
	Test	RMSE	7.5264	**6.7677**	7.3603	7.0452	6.8604	8.4566
		*R* ^2^	0.2963	**0.4310**	0.3271	0.3834	0.4154	0.1116
SO_2_	Train	RMSE	3.2502	**2.9672**	3.2161	2.9913	3.1437	2.4698
		*R* ^2^	0.0453	**0.2044**	0.0652	0.1914	0.1069	0.4487
	Test	RMSE	1.2214	**1.0506**	1.2186	1.1048	1.0574	1.0513
		*R* ^2^	0.2838	**0.4702**	0.2872	0.4140	0.4632	0.4694

*The best results are in bold*.

Additionally, the predicted performance of the models for O_3_ (*R*^2^= 0.8628), CO (*R*^2^= 0.7148) and NO_2_ (*R*^2^= 0.5772) is better than SO_2_ (*R*^2^= 0.4702) and PM_2.5_ (*R*^2^= 0.4310). This can be affected by the distributions and outliers of the data in Figure 2. The outliers in training data are more than the testing data with all pollutants. Especially, these outliers are much higher in SO_2_ and PM_2.5_ than other pollutants. Although *R*^2^ of SO_2_ and PM_2.5_ is under 0.5, the RMSE of these pollutants with LSTM architecture is also lower than other algorithms during the testing period. This performance suggests that the WNMs are reliable in estimating the BAU and, consequently, can provide accurate quantification of the air pollution change during the lockdowns.

It is to note that the errors in the training set are higher than the errors in the test set. Besides the outliers on the data as mentioned above, this can be caused by the “Drop Out” layer and Early Stopping strategy in DL models. On one hand, the “Drop Out” of the TensorFlow library helps the model to reduce the overfitting on the training data, but it is not active in the testing phase. Therefore, the testing error is lower than the training error in some situations. On the other hand, the Early Stopping strategy selects the best parameter setting with the lowest testing error (Fathi and Shoja, 2018).

### Variable Importance

**Figure 6:**
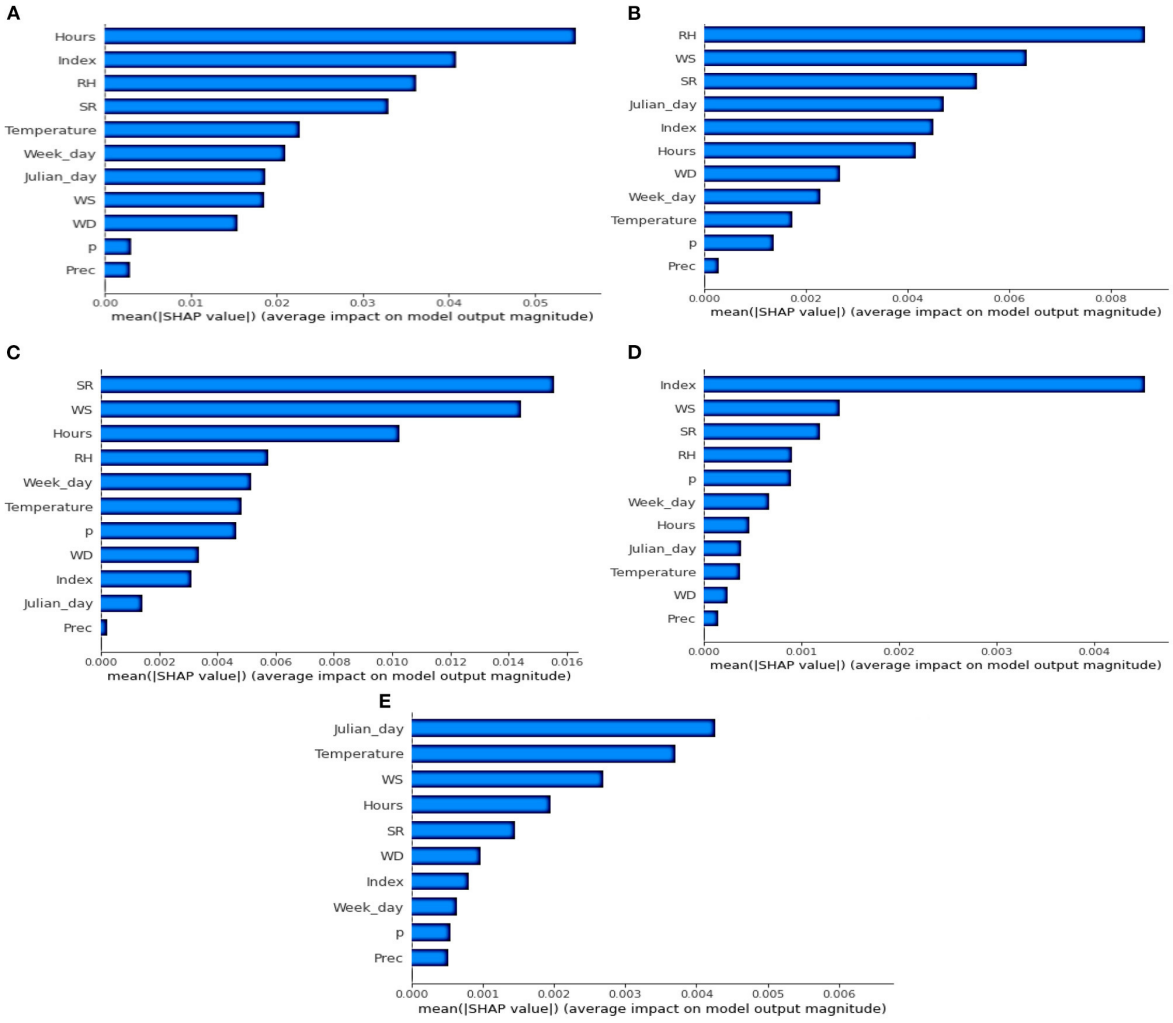
Variable importance with mean (|SHAP value|) for the five best models. **(A)** O_3_ **(B)** CO **(C)** NO_2_ **(D)** SO_2_ **(E)** PM2.5.

SHAP provided an in-depth method for analyzing the contribution of each feature to predict pollutant concentrations in the ML and DL models. Figure 6 shows the mean of SHAP values of the input features with five best models for O_3_ (Figure 6A), CO (Figure 6B), NO_2_ (Figure 6C), SO_2_ (Figure 6D) and PM_2.5_ (Figure 6E). The higher SHAP value means that the feature is more important in predicting the outputs. If we consider the top four variables, both meteorological and temporal features contributed to the best models. Hours, Index, RH and SR, are more important than other variables in the Ozone estimation model (Figure 6A). Ozone is a secondary pollutant that is issued from photochemical reactions, confirming the importance of SR and RH in the concentrations of this feature. Secondly, while SR, WS and Hours have crucial contributions to NO_2_ models (Figure 6C), Index, WS and SR play an important role in predicting SO_2_ concentrations (Figure 6D). WS is a significant feature in most of the best WNMs. This is due to the fact that the wind tends to clean the atmosphere through a ventilation effect. RH is also a good predictor of four pollutants NO_2_, O_3_, SO_2_, and CO. The NO_2_ and CO concentrations are emitted from motorized vehicles and RH tends to worsen engine efficiency, especially in high altitude cities (Zalakeviciute et al., 2018a). Thirdly, the Hours feature was the most significant variable in estimating NO_2_, O_3_, and PM_2.5_ concentrations. It can be explained by the existence of two significant concentration peaks at the rush hours (around 8:00 a.m. and 6:00 p.m.) in the city (Rybarczyk and Zalakeviciute, 2018). The SHAP values of “Hours” are over 0.05 in O_3_, around 0.01 in NO_2_ and over 0.002 in PM_2.5_. Finally, the Julian Day (seasonal) feature is the highest contributor for the PM_2.5_ model (SHAP value ≈ 0.0042). Since the PM_2.5_ emitted from traffic is very sensitive to humidity, the existence of dry and wet seasons can explain the importance of “Julian Day” in the prediction of these latter pollutants (Kleine Deters et al., 2017).

### Quantifying Air Quality Changes

**Figure 7:**
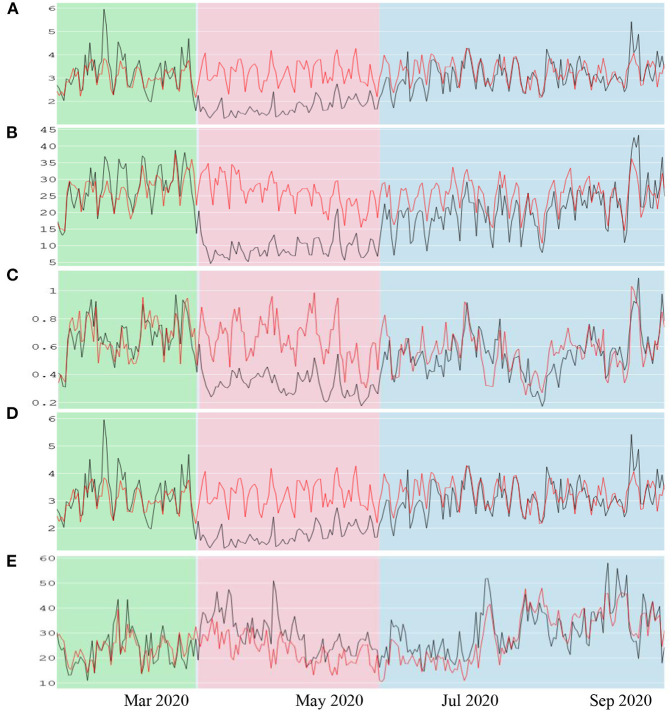
Observed and predicted concentrations of pollutants during the pre-lockdown (green area or validation period), full lockdown (pink area) and partial relaxation (blue area). The black and red lines are the observed and modeled values, respectively. **(A)** represents PM_2.5_ concentrations. **(B)** represents NO_2_ concentrations. **(C)** represents CO concentrations. **(D)** represents SO_2_ concentrations. **(E)** represents O_3_ concentrations.

Figure 7 shows the concentration of PM_2.5_, NO_2_, CO, SO_2_, and O_3_ from 16 January 2020 to 30 September 2020. For the 2 months before lockdown (green area represents the model evaluation period), the estimated values are closer to observations. This confirms that the best models (LSTM for PM_2.5_, SO_2_ and BiRNN for O_3_, NO_2_, CO) provided accurate referential values for quantifying the concentration of contaminants without lockdown (BAU scenario). On the contrary, the concentration of pollutants decreases drastically during the lockdown period (red area in Figure 7). The largest drop, −63.98%, is observed for NO_2_ (Table 5). Meanwhile, CO concentration is reduced by −48.75%. The decline is a bit lower for PM_2.5_ and SO_2_ with −42.17 and −45.76%, respectively. These improvements in air quality can be explained by the substantial reduction in the use of public and private transportations. These results are in line with a previous study (Rybarczyk and Zalakeviciute, 2021), showing that traffic is the main source of pollution in the city center of Quito.

**Table 5:** The effects of two levels of restriction on five pollutants concentrations.

	**PM_**2.5**_**	**NO_**2**_**	**CO**	**SO_**2**_**	**O_**3**_**
Full Lockdown	−42.17%	−63.98%	−48.75%	−45.76%	26.54%
Partial lockdown	−27.22%	−14.95%	−3.52%	−4.84%	6.55%

In contrast to the other pollutants, the concentration of the secondary pollutant, O_3_, increased by 26.54% during the full lockdown. This is due to the weakness of NO_x_-O_3_ titration process (Cazorla et al., 2021). When the concentration of NO_x_/NO_2_ decreased, the concentration of O_3_ tends to increase because of the higher ozone production rates. This is a common urban effect noticed during weekends when anthropogenic activities decrease (Huryn and Gough, 2014).

Finally, during the partial lockdown, the pollutant concentrations displayed in the blue area shows that the BAU values tend to overlap with the observed concentrations. As shown in Table 5, the difference of pollutant concentrations between the actual and predicted values during the partial lockdown is significantly reduced, especially for O_3_ (6.55%), CO (−3.52%), and SO_2_ (−4.84%). This is due to the gradual intensification of anthropogenic activities as businesses started opening up, and more and more people started circulating by using their motorized fleets.

## Conclusions and Future Work

In this research, we proposed WNMs based on DL for quantifying the air quality changes during the full and partial lockdowns due to the COVID-19 pandemic in Quito, Ecuador. In the context of the BAU conditions, the results indicated that DL models are appropriate for assessing the changes in anthropogenic pollutant concentrations. DL is more accurate in predicting the concentration of contaminants than the ML algorithms used in previous studies, namely Random Forest (Lovrić et al., 2021) and GBM (Petetin et al., 2020; Rybarczyk and Zalakeviciute, 2021). Among the DL algorithms, the LSTM and BiRNN are the best architectures for simulating the BAU conditions and can be considered as a promising standard method for assessing air quality.

The study has also demonstrated that our WNMs can capture the correlations among meteorological and temporal variables on five contaminants before the COVID-19 lockdown period. SHAP library allows us to look into the DL black-box and estimate the weights of the input features in the final models. This additional analysis shows that both meteorological and temporal features are relevant in developing the best WNMs. Among the top four variables, we can identify WS, SR and RH for meteorological features and Hours, Index, Julian Day for temporal features.

Our study shows that the concentration of the pollutants decreased by 63.98, 48.75, 45.76, and 42.17% for NO_2_, CO, SO_2_, and PM_2.5_, respectively, during the full lockdown. An increase in O_3_ concentration (26.54%) was attributed to the decline in the NO_2_ concentrations, also known as the weekend effect. On the other hand, as soon as the partial lockdown was implemented and the relaxed regulations on license plate-based circulation were introduced, the pollution concentrations increased gradually up to the BAU level. This fact suggests a short inertia between the alteration of the human mobility and its impact on the air quality of the city.

Even if DL provides better performance than ML, this study is still facing some limitations. First, we do not apply any transformation (e.g., normalization) and remove outliers from the dataset, considering the similar distribution between training and testing sets. However, outliers and skewed data can affect the performance of our models. Second, the error rate is higher in the training set than in the testing set, which suggests further tuning for the Drop Out rate and Patience parameters of DL models. Finally, more advanced algorithms seem necessary to improve the prediction of SO_2_ and PM_2.5_ (*R*^2^ <0.5). Therefore, future work will focus on overcoming these limitations, such as developing WNMs that combine several DL architectures (i.e., LSTM+BiRNN or LSTM+GRU), and normalizing PM_2.5_ and SO_2_ concentrations.

To sum up, the outcome of the present study highlights the fact that air quality in Quito is directly and highly dependent on the mobility of its population. It suggests further research to explore and understand the correlations between traffic, anthropogenic activities, and air pollution. Finally, the general contribution of this work is to propose a new method, DL-based WNMs, allowing an accurate assessment of the effect of any natural event or human intervention on air quality.

## Data Availability Statement

The raw data supporting the conclusions of this article will be made available by the authors, without undue reservation.

## Author Contributions

PC and YR contributed to the conception and design of the study. RZ organized the database. PC implemented the codes, re-processed data and experiments, and wrote the first draft of the manuscript. PC, YR, RZ, and IT contributed to manuscript revision, read, edit, and approved the submitted version. All authors contributed to the article and approved the submitted version.

## Conflict of Interest

The authors declare that the research was conducted in the absence of any commercial or financial relationships that could be construed as a potential conflict of interest.

## Publisher's Note

All claims expressed in this article are solely those of the authors and do not necessarily represent those of their affiliated organizations, or those of the publisher, the editors and the reviewers. Any product that may be evaluated in this article, or claim that may be made by its manufacturer, is not guaranteed or endorsed by the publisher.

## Footnotes

^1^ https://ourworldindata.org/grapher/covid-stringency-index?tab=chart&country=~ECU

^2^ https://github.com/slundberg/shap
